# Techniques in Endovascular Aneurysm Repair

**DOI:** 10.1155/2011/964250

**Published:** 2011-10-25

**Authors:** Sachin V. Phade, Manuel Garcia-Toca, Melina R. Kibbe

**Affiliations:** ^1^Division of Vascular Surgery, University of Tennessee at Chattanooga, 979 East Third Street, Suite C-300, Chattanooga, TN 37404, USA; ^2^Division of Vascular Surgery, Brown University, Two Dudley Street, Suite 470, Providence, RI 02905, USA; ^3^Division of Vascular Surgery, Northwestern University and Jesse Brown Veterans Affairs Medical Center, 676 N. Saint Clair Street, Suite 650, Chicago, IL 60611, USA

## Abstract

Endovascular repair of infrarenal abdominal aortic aneurysms (EVARs) has revolutionized the treatment of aortic aneurysms, with over half of elective abdominal aortic aneurysm repairs performed endoluminally each year. Since the first endografts were placed two decades ago, many changes have been made in graft design, operative technique, and management of complications. This paper summarizes modern endovascular grafts, considerations in preoperative planning, and EVAR techniques. Specific areas that are addressed include endograft selection, arterial access, sheath delivery, aortic branch management, graft deployment, intravascular ultrasonography, pressure sensors, management of endoleaks and compressed limbs, and exit strategies.

## 1. Introduction

Aneurysm repair is a mechanical solution to the problem of progressive expansion of an abdominal aortic aneurysm. Although open surgical repair is very effective, it carries substantial risks from the extensive surgical procedure. Endovascular repair was developed to reduce the risks associated with open surgery and to provide an alternative treatment option for patients who were not good open surgical candidates [1]. Since the first endografts were placed two decades ago, many changes have been made in graft design, operative technique, and management of complications. Herein is a summary of modern endovascular grafts commercially available within the United States, vascular access approaches, methods to visualize relevant anatomy, and techniques to advance large devices, address aortic branches, cannulate the contralateral gate, monitor sac pressures, manage endoleaks, and exit upon completion.

## 2. Endograft Selection

Successful aneurysm exclusion necessitates an understanding of the intricacies of the available grafts for proper preoperative planning and device selection. At present, the Cook Zenith Flex, Endologix Powerlink, Gore Excluder, Gore C3, Medtronic AneuRx, Medtronic Talent, and Medtronic Endurant are the only Food and Drug Administration-(FDA-) approved bifurcated devices available for use in the United States for primary aneurysm repair, while the Cook Renu Converter and Medtronic Talent Converter are approved for secondary conversion to an aorto-uni-iliac device. Characteristics of these endografts are listed in Tables 1 and 2, and several caveats to device delivery are noted in the section on graft deployment.

## 3. Access

Traditionally, femoral access has been obtained by common femoral artery puncture under direct visualization after open exposure. As with open reconstructions vertical or oblique skin incisions may be employed to expose the common femoral artery. Regardless of the incision, the fascia is incised vertically to facilitate arterial dissection. While both incisions allow optimal selection of the arterial puncture site, the former allows easy extension of the incision for urgent iliac artery or infrainguinal revascularization. The latter, meanwhile, is believed to have lower morbidity by some. In a prospective randomized trial of patients undergoing vascular procedures who had no prior surgeries in the index groins, Swinnen et al. demonstrated a lower complication rate with transverse incisions (47.5% versus 12.7%, *P* < 0.001) [2]. There were 13 (11%) wound infections in 116 groins by postoperative day 28, with 3 in patients with transverse incisions and 10 in patients with vertical incisions (*P* = 0.062). Lymphatic leaks were present in 12.7% of those with transverse incisions, as opposed to 27.9% of wounds with vertical incisions (*P* = 0.044). Of note, while the authors from this study observed a difference favoring transverse incisions, their overall wound complication rate was substantially higher than those reported by other series of femoral exposures for EVAR, which range from 2 to 2.8% [3, 4].

Percutaneous femoral access has been reported with two suture-mediated “preclose” techniques, using either the Abbott ProStar XL or Abbott Perclose ProGlide. Both techniques involve blind or ultrasound-guided percutaneous access using micropuncture or standard access needles. With the micropuncture technique, the entry needle is exchanged for a 4 French (Fr) sheath, through which ipsilateral oblique angiography is performed to confirm common femoral artery access. When a standard entry needle is used, the needle should still be replaced with a sheath to confirm optimal anterior wall access in the common femoral artery before proceeding. Regardless of the entry technique, a 0.035 inch wire is advanced, the sheath is withdrawn, and a hemostat is used to dilate the entire tract to prepare it for introduction of the large endovascular devices. If the ProStar XL 10 Fr closure device is used, the device is advanced into the arteriotomy until pulsatile bleeding is noted from the marker port; the two sutures are then deployed, retrieved, and left untied. The closure device is then exchanged for a large sheath or the endograft device over a stiff wire. The same technique can be used for the contralateral side. If the Perclose ProGlide 6 Fr closure device is used, it is important to deploy the two devices at 90 degrees from each other (i.e., 45 degrees from the midline in each direction) so as to achieve optimal closure of the large arteriotomy with the two sutures. Once pulsatile bleeding is obtained from the marker port of the first device, the suture is deployed, retrieved, and left untied yet secured. The closure device is then exchanged for the second ProGlide device over a 0.035^'^ wire, which is rotated 45 degrees from the midline in the opposite direction, deployed with the sutures left untied yet secured, and exchanged for the endograft device or a large sheath over a stiff wire. Similarly, this technique can be used for the contralateral side. In patients with tortuous or stenotic iliac arteries, a combination of angled catheters and wires may be required to gain enough wire purchase for safe exchanges. Regardless of the percutaneous technique used, the sutures are secured on shod clamps, ensuring no tension on the sutures during the case. The sutures and clamp may be covered with a damp sterile towel to ensure they do not become entangled in the subsequent wires, catheters, and endograft.

## 4. Angiography

Angiography is an essential diagnostic tool for EVAR. Typically performed with low osmolar, nonionic agents, angiography is the standard method of obtaining landmarks for graft deployment and ensuring adequate aneurysm exclusion. Digital subtraction angiography, specifically, allows optimal visualization of the contrast. Often, preoperative imaging can be used to guide wire, catheter, and graft placement, thereby limiting intravenous contrast exposure. The contrast may also be diluted to reduce the risk of contrast-induced nephropathy. Alternatively, carbon dioxide may be substituted as the contrast agent.

## 5. Sheath Delivery

Currently available endografts require large femoral and iliac artery vessels to accommodate the ipsilateral and contralateral limb devices. While a combination of angled catheters, angled wires, hydrophilic wires, and stiff wires may be used to track the sheaths proximally, three options are available when hypoplastic, stenotic, occluded, or tortuous vessels preclude traditional femoral access for device delivery. This includes direct puncture, use of a conduit, and controlled dilation or rupture of the artery.

First, the aorta and iliac arteries may be directly punctured proximal to any significant stenoses to allow device delivery. This may be done via a retroperitoneal approach with minimal dissection of the proposed access vessels. Without the need for circumferential arterial control, two purse string sutures can be placed such that the sutures are begun and ended 180 degrees away from each other. A needle should be used to obtain access in the center of the two sutures, which can be controlled by the operator or assistant during sheath exchanges and should facilitate easy closure of the artery [5].

Second, retroperitoneal exposure can allow placement of a prosthetic conduit, sutured to the aortoiliac system, as dictated by preoperative computed tomographic angiography (CTA). Via a standard lower quadrant oblique incision, the distal aorta or common iliac artery may be exposed to allow an end-to-side anastomosis to be performed with a 10 mm prosthesis of the surgeon's preference. Placement of the conduit at a sharp angle with the native artery should be avoided; the conduit can be tunneled along the native artery to the groin to blunt the angle of approach. The suture line may be reinforced with graft material to reduce bleeding, and manual guidance of the sheath can minimize anastomotic disruption. A radiopaque marker such as a clip can also be placed at the anastomosis to facilitate delivery sheath placement distal to the anastomosis and reduce exchanges across the suture line. With the terminal portion of the conduit ligated, the side wall of the conduit is punctured for the diagnostic catheter and device delivery sheaths [6]. Alternatively, the device delivery sheath and an additional small sheath may be placed through the end of the conduit, with vessel loops for hemostasis, allowing both device introduction and placement of a diagnostic angiography catheter. 

**Figure 1:**
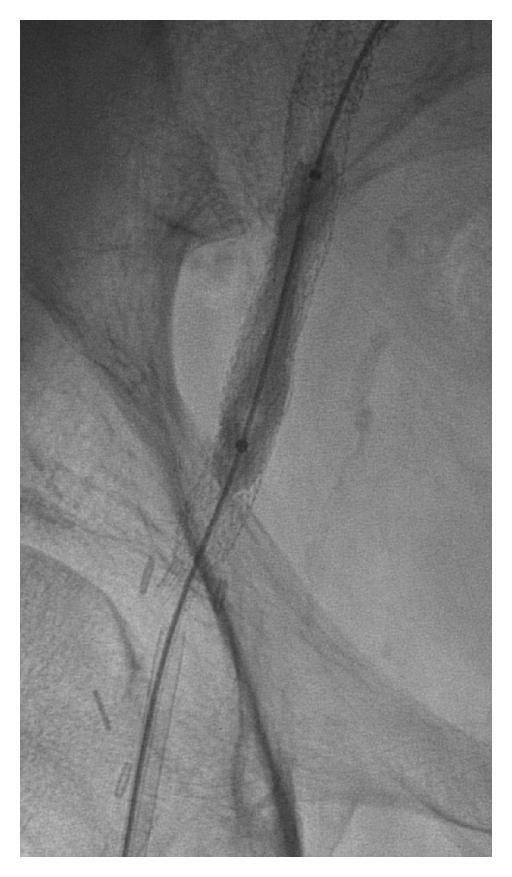
Controlled ruptured of the iliac artery with balloon angioplasty of a covered stent.

Third, a variety of techniques can be used to dilate the iliac arteries and avoid retroperitoneal exposure. Primary iliac artery balloon angioplasty may suffice to allow passage of many sheaths. Alternatively, serial dilation with hydrophilic dilators may be employed. Another technique described by von Segesser et al. involves insertion of a sheath smaller than what would be required for device delivery; afterwards, in situ balloon dilation of the sheath is performed to limit iliac artery injury and allow passage of the device [7]. Yet another method involves creating a controlled dilation or rupture of the iliac artery with a large covered stent to allow for larger sheath delivery through the “endoconduit” [6]. Most often, the internal iliac artery must be covered with this method, and pelvic ischemia can result. Endograft limbs may be used, but the Gore Viabahn, Bard Fluency plus vascular stent graft, and Atrium iCAST covered stents could also be used off label for this technique (Figure 1). While both the Viabahn and Fluency stents are self-expanding covered stents available in 5 mm to 13 mm diameters, the latter has uncovered stents proximally and distally. In contrast, the iCAST stent provides a balloon expandable option in 5 mm to 10 mm diameters.

## 6. Management of Large Branches

There are few large aortoiliac branches that must be addressed during endovascular repairs of infrarenal abdominal aortic aneurysms. These branches include lumbar arteries, the inferior mesenteric artery, internal iliac arteries, and accessory renal arteries, and they may be addressed by occlusion or preservation.

Although most type II endoleaks in the setting of stable aneurysms are currently treated expectantly, there is some controversy regarding preoperative and intraoperative branch occlusion [8]. Patent inferior mesenteric arteries and large lumbar arteries, which are potential sources of type II endoleaks, may be occluded; likewise, hypogastric arteries that must be covered for an adequate distal seal may be occluded. Typically, transarterial embolization is performed with a combination of platinum and stainless steel coils placed at the origin of these branches to occlude the orifice yet preserve collateral flow. With the “anchoring technique,” a catheter is placed in a smaller branch of the vessel in question; as the coil is released, the catheter and coil are pulled back into the larger branch close to its origin. This allows fixation of the coil with proximal branch embolization. Meanwhile, with the “scaffolding technique,” a large coil is placed at the branch vessel origin and smaller coils are packed within the large coil to avoid distal embolization. The Amplatzer plug, available in 3–22 mm sizes, is another device used for transarterial embolization and allows controlled occlusion of both small and large branches. When selecting an embolization device, both coils and plugs should be oversized with relation to the index vessel. In fact, unlike other devices, the Amplatzer occluder instructions for use call for oversizing of 30–50%. The coils can provide flexibility in tortuous or tight areas, while the plugs allow accurate deployment in short landing zones. If both internal iliac arteries must be occluded, staged embolization may allow collateral formation and reduce pelvic ischemia. High-viscosity Onyx mixed with contrast agents has been used for postoperative embolization of clinically significant type II endoleaks but is not typically used during the index procedures as type II endoleaks typically have a benign course and the contrast interferes with postoperative endoleaks surveillance.

More proximally, if faced with a marginal neck length or a significant accessory renal artery that is to be preserved, the “chimney technique” may be used to accomplish endovascular aortic aneurysm repair. Via a brachial or axillary approach, the renal artery in question is accessed. A covered stent is then deployed, with the stent extending into the aorta in a cranial direction. A standard EVAR is then performed, with care to dilate the renal artery conduit and aortic graft simultaneously to preserve renal perfusion and achieve an adequate proximal aortic seal [9]. 

If the distal limbs must be extended into the external iliac arteries, the risk of pelvic ischemia and paralysis increases. Internal iliac arteries may be preserved with a “snorkel technique,” but like its proximal counterpart, the long-term results are unknown. The internal iliac artery can be accessed through a brachial, axillary, ipsilateral, or contralateral femoral approach, depending upon the anatomy and angulation of the internal iliac artery with respect to the common and external iliac artery. Wire and catheter exchanges are performed, as necessary, to enable sheath advancement for covered stent placement. A standard iliac extension is then performed into the external iliac artery via ipsilateral femoral access. “Kissing balloons” are inflated in both the internal and external iliac limbs to fully expand the grafts.

Custom-made fenestrated and branched endografts may also be employed to preserve larger aortic branches, including but not limited to the renal arteries, mesenteric vessels, and internal iliac arteries. However, as deployment is complex, individualized, and currently investigational, technical details are herein omitted.

## 7. Graft Deployment

While the graft deployment steps vary based on the endograft selected, there are many common features and a few differences that are worthy of mention. First, determination of the side of main body and contralateral leg delivery is dependent on access vessel diameter, iliac artery tortuosity, main body length, and iliac artery diameter. Clearly, if an iliac artery stenosis is present, delivery of the large main body through the larger side would carry less risk of iliac artery injury. While placement of the main body through an tortuous iliac artery could potentially be difficult, tortuosity could also make wire and catheter management during access of the contralateral gate tricky, as stored energy prevents extracorporeal wire manipulation from being translated into one-to-one intravascular movement. Additionally, as limited combinations of aortic diameters, iliac artery diameters, and endograft lengths exist, device delivery involves matching vessel size with endograft availability.

Second, deployment close to the renal arteries is preferred, but the grafts should be placed within a neck of relatively uniform diameter, with reversed taper necks offering the most challenge. Currently, according to the manufacturer's instructions for use, the Medtronic Talent and Endurant devices are the only devices approved for use with 10 mm long infrarenal necks; other devices require at least 15 mm for the proximal neck length. Mural thrombus along the infrarenal neck and suprarenal portion of the aorta may also guide endograft selection in order to avoid thromboembolic complications, graft migration, or endoleaks. Certainly, wire, catheter, sheath, and device manipulation should be limited in patients with tremendous mural thrombus burden due to thromboembolism. A patient with isolated suprarenal disease may be better suited for a device without a suprarenal stent in order to minimize embolization into the renal arteries or distally. Infrarenal thrombus located along the seal zone of the neck should be approached cautiously, as significant circumferential thrombus burden is a relative contraindication for endograft use. If the thrombus load is mild, suprarenal fixation to healthy aortic tissue should be considered. 

Third, aortic angulation, while usually not a problem, must be considered before graft selection and deployment. The Medtronic AneuRx is contraindicated in aortas with more than 45 degrees of angulation, while all other endografts are contraindicated with necks with more than 60 degrees of angulation. Additionally, the Cook Zenith Flex is not recommended when the suprarenal stent would be located within an aortic segment with more than 45 degrees of angulation.

Next, graft selection and deployment usually require consideration of aortic length, aortic diameter at the level of the contralateral gate, the narrowest aortic diameter, position of the iliac arteries, and the iliac artery length. Currently, the Gore Excluder and Gore C3 have the shortest bodies, allowing the contralateral gate to be opened 70 mm below the top of the covered stents. Most often, however, long main bodies are preferred, as opening the contralateral gate in proximity to the common iliac artery orifice facilitates gate cannulation. Another factor in determining the main body length is the aortic diameter, both at the site of the contralateral gate and the narrowest point, as the diameter must be sufficient to prevent limb constriction. Circumferential calcification along narrow segments is particularly troublesome, as it often prevents graft expansion. Additionally, depending on iliac artery position and length, the main body may be rotated up to 180 degrees to facilitate contralateral gate cannulation or slightly shorten the iliac limbs. Conveniently, the Gore C3 was recently released as the only device that allows a portion of the graft to be recaptured (up to three times) to facilitate repositioning of the proximal graft and contralateral gate. Several programs, such as those of M2S and TeraRecon, are available to help with this planning by constructing three-dimensional imaging and predicting center lines.

Lastly, most of the endoprostheses available in the United States are bifurcated modular grafts that require placement of a contralateral limb. Free-style cannulation of the contralateral gate is most often performed with an angled catheter and wire. When this fails, a selective catheter placed from the ipsilateral limb or upper extremity can be used in combination with a snare to obtain wire access into the contralateral gate. If difficulty with contralateral gate cannulation is anticipated, the Endologix Powerlink graft may be used, as it employs a bifurcated unibody design that is deployed, pulled down onto the aortic bifurcation, and then modified with proximal and distal extensions, if needed.

## 8. Intravascular Ultrasonography

While conventional angiography is essential for endovascular aneurysm repair, intravascular ultrasonography (IVUS) can be a helpful adjunct for graft selection, deployment, and further interrogation. For most aortic procedures, 8 to 15 MHz transducers are selected, with lower frequencies offering the ability to view the entire artery while sacrificing resolution. After obtaining access to both groins, the IVUS catheter can be used to mark the location of the renal arteries aortic bifurcation, and hypogastric arteries, and measure the diameters of the proximal aorta, aortic bifurcation, and iliac arteries. If performed over a flexible wire, proximal neck and iliac artery lengths can be determined before a stiff wire alters the anatomy. With this, contrast angiography and significant fluoroscopy can be avoided until the main body is positioned for deployment. The contralateral gate can then be cannulated with IVUS, further reducing radiation exposure and contrast administration. Finally, the entire endograft can be interrogated for endoleaks and apposition to the arterial wall [10].

## 9. Pressure Sensors

Computed tomographic angiography is currently the gold standard for EVAR surveillance. The instructions for use for most devices suggest postoperative imaging at 1, 6, and 12 months during the first year and annually thereafter, if the patient remains asymptomatic and no other concerning findings on physical examination or prior imaging exist. The IFU for the Medtronic Endurant graft is the first to suggest less frequent surveillance, with imaging recommended at 1 and 12 months and then yearly thereafter. However, in order to reduce contrast and radiation exposure, investigations are being performed on noninvasive remote aneurysm sac pressure measurements, primarily using the CardioMems Endosure Wireless AAA Pressure Sensor. This device, which is about the size of a paperclip, requires transcatheter placement through a 14 Fr delivery device into the aneurysm sac after main body deployment. The sensor is interrogated, the contralateral limb is deployed, the sensor pressures are measured again, and then the sensor is finally deployed. Despite the paucity of evidence demonstrating device durability and efficacy, preliminary studies have demonstrated promising short-term results [11–13].

## 10. Assessment and Management of Early Endoleaks and Compressed Limbs

After the graft deployment and inflation of a molding balloon, digital subtraction angiography or IVUS is used to assess for endoleaks. Some type I endoleaks can be managed with simple angioplasty or proximal or distal graft extensions, when possible. Additionally, bare stents can be used to juxtapose the endograft to the arterial wall. Most early type II endoleaks are managed with initial observation, with prompt return for reintervention for those who are symptomatic or who have enlargement of the aneurysm sac. Type III endoleaks can be treated by relining the segment of graft fracture or separation. Mild external compression of the contralateral limb may be treated with angioplasty, with selective stent placement. Alternatively, compressed limbs, inaccessible contralateral gates, and persistent endoleaks can sometimes be treated with a hybrid conversion, combining an aorto-uni-iliac converter device (Table 2) with a femoro-femoral bypass. Finally, for those complications that cannot be managed or fail observation, endovascular, or hybrid management, open conversion with explantation of the endoprosthesis and traditional open repair can be performed.

## 11. Exit Strategy

**Figure 2:**
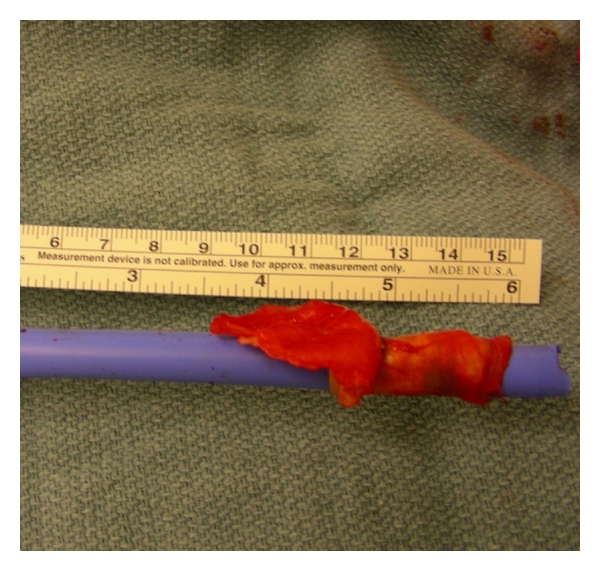
Photograph of a portion of the iliac artery that was withdrawn with the sheath, otherwise known as “iliac-on-a-stick.”

After confirming that no significant endoleaks or flow-limiting stenoses are present, the delivery devices and sheaths should be withdrawn while maintaining wire access. If a precipitous change in hemodynamics occurs or a segment of artery is withdrawn with the sheath, otherwise known as “iliac-on-a-stick” (Figure 2), catheters can readily be passed for diagnostic imaging and occlusive balloons can be inflated for hemostasis. If needed, arterial injuries can then be addressed under a more controlled situation.

Arteriotomy closure depends on the initial approach for access. If femoral access was obtained via a traditional open approach, a standard arteriotomy repair should be performed, with subsequent layered closure of the wound. Alternatively, if the “preclose” technique was utilized, manual pressure is maintained proximally while the wounds and sutures are rinsed free of thrombus and the tract is confirmed to be fully dilated. The sutures are then tied, with the knots carefully pushed down to, but not into, the artery. The wire should be removed before tying the second suture. Despite successful closure, a brief period of manual compression may be required. Emergent femoral artery repair should be performed if significant hemorrhage persists or extremity ischemia develops. However, several authors have reported a low incidence of short- and midterm complications using the “preclose” technique [14–17]. Specifically, technically successful percutaneous closure can be performed in approximately 95% of access sites, and mid- to long-term followup has demonstrated few pseudoaneurysms and hemodynamically significant stenoses. As expected, extensive calcification, groin scarring, and operator inexperience increase the risk of complications. If a conduit is sutured to the aorta or iliac arteries, it may be ligated and transected proximally or preserved for an aortofemoral or iliofemoral bypass. Alternatively, the conduit can be conserved for later re-intervention by the subcutaneous placement of the ligated end. Via a small incision, this “buried treasure,” as referred to by Jon Matsumura (personal communication), can later be accessed after thrombectomy for future interventions.

Lastly, a lower extremity pulse examination should be performed to exclude acute ischemic changes. Ankle-brachial indices and Doppler signals may be obtained on the operating table and compared to preoperative assessments as objective adjuncts to the clinical examination. Any significant issues can thereby be addressed immediately.

## 12. Conclusion

Endovascular aneurysm repairs require extensive preoperative preparation not only for graft selection but also for optimal deployment. One must consider the method of access, device delivery, management of large branches, graft orientation, and contralateral limb deployment. Once deployed, the graft must be assessed for endoleaks and limb compression before arteriotomy and wound closure. Failure to understand these EVAR techniques and adequately plan for each step can result in endoleaks, visceral malperfusion, and extremity ischemia, precipitating the need for emergent complex open repair.

## Figures and Tables

**Table 1:** FDA approved devices for primary infrarenal aortic aneurysm repair.

Device	CT measurement method^(1)^	Delivery sheath required	Minimum access diameter for main body	Minimum access diameter for contralateral limb	Treatable aortic neck diameter	Minimum aortic length to bifurcation	Minimum aortic neck length	Maximum aortic angulation^(2)^	Suprarenal fixation	Treatable iliac diameter	Minimum iliac seal zone
Cook Zenith Flex	Outer diameter	No	22–26 mm graft = 7.1 mm 28–32 mm graft = 7.7 mm 36 mm graft = 8.5 mm	8–10 mm graft = 5.3 mm 12–24 mm graft = 6.0 mm	18–32 mm	22–32 mm graft = 82 mm 36 mm graft = 95 mm	15 mm	<60 degrees, <45 degrees^(3)^	Mandatory	7.5–20 mm	10 mm

Endologix Powerlink	Outer diameter	No	7 mm	3 mm	18–32 mm	25–28 mm graft = 80 mm 34mm graft = 100 mm	15 mm	<60 degrees	Available	10–23 mm	15 mm

Gore Excluder	Inner diameter	Yes	23–8.5 mm graft = 6.8 mm 31 mm graft = 7.6 mm	12–14.5 mm graft = 4.7 mm 16–20 mm graft = 6.8 mm	19–29 mm	70 mm	15 mm	<60 degrees	Not available	10–18.5 mm	10 mm

Gore C3	Inner diameter	Yes	23–8.5 mm graft = 6.8 mm 31 mm graft = 7.6 mm	12–14.5 mm graft = 4.7 mm 16–20 mm graft = 6.8 mm	19–29 mm	70 mm	15 mm	<60 degrees	Not available	10–18.5 mm	10 mm

Medtronic AneuRx	Outer diameter	No	7 mm	5 mm	16–26 mm	80 mm	15 mm	<45 degrees	Not available	10–22 mm	25 mm

Medtronic Endurant	Inner diameter	No	23–25 mm graft = 5.7 mm 28–36 mm graft = 6.4 mm	10–16 mm graft = 4.5 mm 20–28 mm graft = 5.1 mm	19–32 mm	124 mm length graft = 74 mm 145–166 mm length grafts = 84 mm	10 mm	≤60 degrees	Mandatory	8–25 mm	15 mm

Medtronic Talent	Outer diameter	No	22–28 mm graft = 7 mm 30–36 mm graft = 8 mm	6 mm	18–32 mm	80 mm	10 mm	<60 degrees	Available	8–22 mm	25 mm

^(1)^Based on Instructions for Use (IFU).

^(2)^Angulation of neck relative to long axis of aorta.

^(3)^Angulation of suprarenal stents relative to long axis of aorta.

**Table 2:** FDA-approved devices for secondary intervention after EVAR.

Device	CT measurement method	Delivery sheath required	Minimum access diameter for main body	Treatable aortic neck diameter	Minimum aortic length to bifurcation	Minimum aortic neck length	Maximum aortic angulation^ (1)^	Suprarenal fixation	Treatable iliac diameter	Minimum iliac seal zone
Cook Renu Converter	Outer diameter	No	22–26 mm graft = 7.1 mm 28–32 mm graft = 7.7 mm 36 mm graft = 8.5 mm	18–32 mm^ (2)^	22–32 mm graft = 82 mm 36 mm graft = 95 mm	15 mm^ (3)^	<60 degrees, <45 degrees^ (4)^	Mandatory	7.5–20 mm	10 mm

Medtronic Talent	Outer diameter	No	22–28 mm graft = 7 mm 30–36 mm graft = 8 mm	18–32 mm	80 mm	10 mm	<60 degrees	Available	8–22 mm	25 mm

^(1)^Angulation of neck relative to long axis of aorta.

^(2)^New graft must be oversized 2 mm if placed within a preexisting graft.

^(3)^Neck must be ≥10 mm if new graft is placed within a preexisting graft.

^(4)^Angulation of suprarenal stents relative to long axis of aorta.
